# Integrated cellulase continuous production and downstream processing using a packed-bed bioreactor for solid-state fermentation by thermophilic fungus

**DOI:** 10.1007/s00449-026-03371-1

**Published:** 2026-06-29

**Authors:** Nilton S. C. Mafra, Fernanda P. Casciatori

**Affiliations:** 1 Graduate Program of Chemical Engineering, Federal University of São Carlos, Rod. Washington Luís km 235—SP-310, São Carlos, SP 13565-905 Brazil; 2 Chemical Engineering Department, Federal University of São Carlos, Rod. Washington Luís km 235—SP-310, São Carlos, SP 13565-905 Brazil

**Keywords:** Solid-state cultivation, Enzyme recovery, Thermal control, Cellulase production, Second-generation ethanol

## Abstract

In situ cellulase production is a key strategy to enhance efficiency, sustainability, and robustness of second-generation bioethanol processes. However, limitations related to heat transfer, bioreactor operation, and downstream processing still hinder large-scale implementation of solid-state fermentation (SSF) for enzymes production. This study systematically evaluated the integrated production, extraction, recovery and stabilization of endoglucanase from the cultivation of *Myceliophthora thermophila* I-1D3b in a multilayer packed-bed bioreactor operated under batch and continuous modes, using sugarcane bagasse and wheat bran (7:3 w/w) as substrate. Fermentations were carried at 45 °C for 96 h, and the enzymatic extracts were characterized as a function of pH and temperature, as well as subjected to concentration by ammonium sulfate and ethanol precipitation. Maximum enzymatic activity was achieved at 60 °C and pH 4.0, confirming a thermoacidophilic profile suitable for industrial applications. Ammonium sulfate precipitation outperformed ethanol, achieving 78.7% of recovering of total units of enzymatic activity from the raw extract and an activity of 156 U·g.d.s⁻¹ (enzyme units per gram of dry substrate), whereas ethanol resulted in significant activity losses under the evaluated conditions. Within the bioreactor, maximum temperatures reached 48.2 °C (batch) and 48.9 °C (continuous), indicating effective mitigation of overheating. Increasing percolation water flow rate significantly enhanced enzyme recovery, highlighting mass transfer as a key limiting factor in downstream performance. Similar average activities were obtained for batch (95.63 ± 28.37 U·g.d.s⁻¹) and continuous operation (96.36 ± 4.63 U·g.d.s⁻¹), while continuous mode reduced variability among modules, indicating improved spatial homogeneity. Overall, process integration combining thermal control, percolation-based extraction, and downstream processing enhances robustness and industrial applicability of SSF-based cellulase production.

**Supplementary Information:**

The online version contains supplementary material available at 10.1007/s00449-026-03371-1.

## Introduction

The circular bioeconomy has been widely recognized as a promising strategy for the sustainable valorization of organic by-products by enabling their conversion into energy, biomaterials, and value-added bioproducts [1]. In this context, lignocellulosic biorefineries have emerged as key platforms by integrating biotechnological and physicochemical routes, thereby improving resource efficiency and mitigating environmental impacts. Among the most relevant applications, second-generation bioethanol (2G ethanol) stands out, whose feasibility strongly depends on the availability of efficient cellulolytic enzymes for lignocellulosic biomass saccharification [2, 3].

In situ cellulase production represents a strategic alternative to reduce reliance on commercial enzyme formulations and to enhance enzymatic self-sufficiency in lignocellulosic biorefineries [4, 5]. Among the available approaches, solid-state fermentation (SSF) has gained increasing attention due to its ability to directly utilize agro-industrial by-products as substrates, while requiring lower water input compared to submerged systems and aligning closely with circular bioeconomy principles [6]. Recent studies highlight SSF as an emerging platform for enzyme and bioproduct generation from such materials, converting secondary streams into valuable inputs for biotechnological processes [7].

Among the bioreactor configurations employed in SSF, packed-bed systems are particularly attractive due to their simple design, low operational cost, and suitability for filamentous fungal growth on solid matrices. However, these systems still face significant limitations related to heat and mass transfer. The low thermal conductivity of the bed and restricted aeration promote the formation of internal temperature and moisture gradients, which can compromise process stability and scalability [8, 9]. To address these limitations, several strategies have been proposed, including multilayer bed configurations and alternative operating modes aimed at mitigating overheating and improving overall system performance [10].

Despite these advances, studies integrating continuous operation in packed bed bioreactors with systematic downstream processing, including percolation-based extraction, enzyme concentration, and stabilization, remain scarce. Rodrigues et al. [10] demonstrated the feasibility of continuous operation using *Myceliophthora thermophila* I-1D3b in a multilayer packed bed system with online CO₂ monitoring. However, integrated strategies for enzyme recovery and concentration throughout the process were not addressed. This gap limits the transition of SSF based processes toward fully integrated platforms for in situ cellulase production. Therefore, the overall efficiency of SSF systems depends not only on fermentation performance but also on the effective integration of upstream and downstream stages, including appropriate enzyme recovery, concentration, and stabilization strategies under process relevant conditions [7, 11, 12].

In this context, this study proposes an integrated process framework for endoglucanase production in solid-state fermentation, combining reactor design, operation strategy, and downstream processing within a single system. A multilayer packed-bed bioreactor operated under batch and continuous modes was used to systematically evaluate the coupling between heat and mass transfer phenomena, enzymatic production, and recovery efficiency. Emphasis was placed on identifying transport limitations during percolation-based extraction and on assessing the impact of operating mode on process homogeneity and stability. In addition, enzyme concentration strategies were investigated to establish a robust downstream route compatible with SSF systems. By integrating upstream and downstream stages and providing insights into reactor operation and process intensification, this work contributes to overcoming key bottlenecks associated with the scale-up and industrial implementation of SSF-based cellulase production.

## Materials and methods

### Microorganism and substrates

The thermophilic fungus *Myceliophthora thermophila* I-1D3b was used as a cellulase-producing microorganism. The strain was originally isolated from sugarcane bagasse piles at Usina Guarani (Olímpia, SP, Brazil) and selected due to its high performance in cellulase production in experiments conducted at flask and bioreactor scales, as previously reported [13].

For inoculum preparation, the strain was cultivated in slant agar cultures in Erlenmeyer flasks containing 60 mL of Sabouraud Dextrose Agar (SDA) medium. The flasks were incubated in a biochemical oxygen demand chamber (BOD) (Model SL 200, Solab, Brazil) at 45 °C for at least 48 h to allow mycelial growth and sporulation. After this period, the agar surface was gently scraped with 100 mL of sterile nutrient solution to remove the spores. This solution, adjusted to pH 5.0, had the following composition (w/v): 0.35% (NH₄)₂SO₄, 0.30% KH₂PO₄, 0.05% MgSO₄·7 H₂O, 0.05% CaCl₂, and 0.10% Tween 20, as described by Zanelato et al. [13]. The spore suspension was standardized to 1 × 10⁷ spores per gram of dry solid (g.d.s).

As solid substrates, sugarcane bagasse (SCB) and wheat bran (WB) were used, selected due to their low cost, high regional availability, and for providing favorable nutritional and structural conditions for the solid matrix [14]. SCB was supplied by Usina Ipiranga Agroindustrial S.A. (Brazil), while WB was obtained from a local supplier (Agropecuária Claro, São Carlos, SP, Brazil). SCB was previously dried in a convection oven (Model 035, Marconi, Brazil) at 60 °C until reaching a moisture content between 10 and 15%, determined using a moisture analyzer (M5 Thermo, Bel Engineering, Monza, Italy). After drying, the material was sieved using mesh sizes of 5 × 5 mm and 4 × 2 mm. Wheat bran was used as received, with moisture content of 12% (w.b.) and average particle sizes up to 1.8 mm.

### Solid-state fermentations in packed-bed bioreactor

**Fig. 1:**
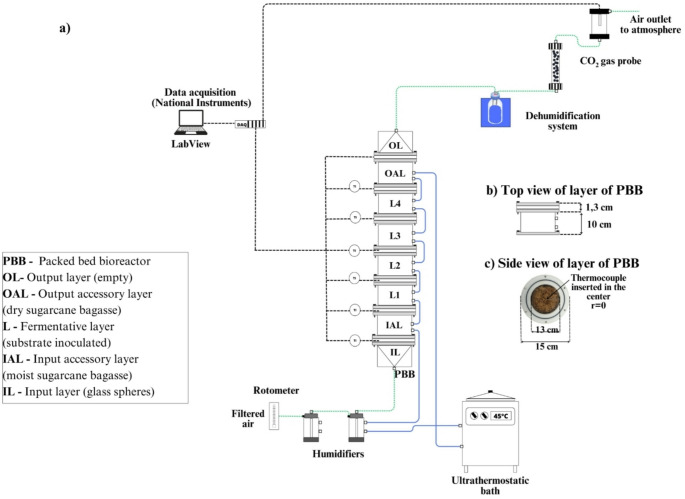
Experimental setup of the packed-bed bioreactor (PBB): **a** operating system, **b** side view of the module, and **c** top view of the module

The SSF experiments were carried out in a packed-bed bioreactor (PBB) described by [10] and illustrated in Fig. 1a. The system, constructed of stainless steel, consisted of cylindrical modules with perforated bottoms, measuring 10 cm in length, 13 cm in internal diameter, and 15 cm in external diameter, as shown in Fig. 1a and b. The annular space of each module served as a water jacket, enabling bed temperature control and mitigating overheating phenomena typically observed in packed-bed SSF.

Process air was supplied by a compressor and passed sequentially through a needle valve, a biological filter, and an AppliTech^®^ flow meter (Asa, Italy), before being directed to a jacketed column filled with glass beads. The void space between the beads was filled with distilled water maintained at the operating temperature to promote air saturation prior to its entry into the bioreactor, a strategy recommended to minimize excessive bed drying during SSF. At the reactor outlet, the percolated gas passed through a dehumidification system consisting of a condensate collector connected to a receiving flask, followed by a silica gel column measuring 5.2 cm in diameter and 39 cm in height. This arrangement reduced the relative humidity of the outlet air to values below 15%, a condition required for proper measurement of CO₂ concentration using a Vaisala Carbocap probe (Vantaa, Finland).

Similarly to the system described by Oliveira et al. [11], the bioreactor was vertically oriented and included inlet (IL) and outlet (OL) modules, both jacketed and with the same external dimensions as the intermediate fermentation modules, but with a conical internal geometry. During operation, air flowed upward through the fixed bed. The inlet module (IL), located at the base of the system, was filled with glass beads to promote homogeneous air-flow distribution, whereas the outlet module (OL) was left empty to facilitate gas flow. In addition, two auxiliary modules containing wet coarse sugarcane bagasse (IAL) and dry coarse sugarcane bagasse (OAL), respectively, were used to reduce excessive water removal from the fermentation modules and to absorb moisture condensed on the walls of the outlet module. Each auxiliary module was filled with 90 g of coarse SCB.

The operating temperature was maintained at 45 °C, which has been reported as optimal for endoglucanase production by *M. thermophila*, as described byRodrigues et al. [10]. The fermentation time was 96 h. During the experiments, both the process air and the circulating water were maintained at 45 °C through water circulation in the jackets of the modules and humidifiers, supplied by a thermostatic bath (model SL-152, Solar, Brazil), while the percolating air flow rate was fixed at 250 L h⁻¹ to enhance oxygen transfer and maintain adequate moisture conditions throughout the bed.

The fermentation temperature profile was monitored over time using T-type thermocouples installed through flanges at the inlet and outlet of each fermentation module. The sensor tips were positioned at the radial center of each module (Fig. 1c), allowing the evolution of the internal bed temperature to be monitored throughout fermentation. The signals were recorded using a National Instruments COMPAQ-DAQ data acquisition system operated with a routine developed in LabVIEW software (National Instruments, Austin, USA).

During the addition, removal, and repositioning of the modules, the solid substrate remained temporarily exposed to the laboratory environment. However, all handling procedures were performed under aseptic conditions to minimize microbial contamination. The substrates and nutrient solution were sterilized separately in an autoclave at 121 °C for 20 min. After cooling, the components were mixed and homogenized with the spore suspension in a laminar-flow cabinet previously sterilized by ultraviolet radiation. The air filters and humidification devices were also sterilized before each fermentation run.

The fermentation medium was prepared using a mixture of SCB and WB at a ratio of 7:3 (w/w), in agreement with previous studies on *M. thermophila* I-1D3b [9]. The moisture content was adjusted to 75% (wet basis) by adding the nutrient solution and the inoculum suspension enough to provide 1 × 10⁷ spores per gram of dry solid. Each fermentation module was filled with 130 g of dry substrate.

#### Operating mode

**Fig. 2:**
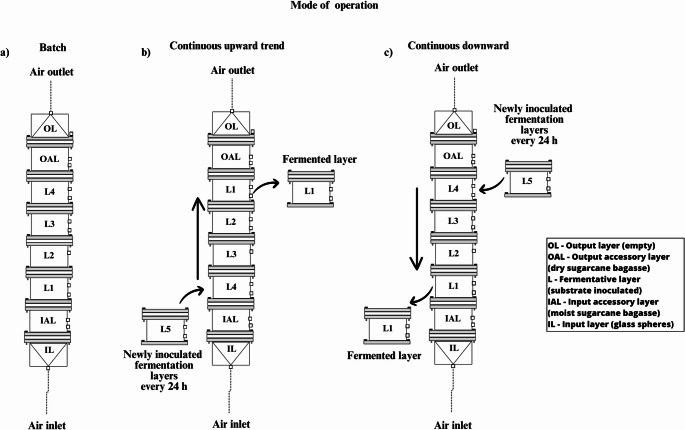
Operational configurations of the packed-bed bioreactor under (**a**) batch; and continuous modes, **b** upward; **c** downward. In continuous operation, modules are sequentially added and removed, allowing simultaneous processing at different fermentation stages

To evaluate the effect of the operating mode (batch and continuous) on bioprocess performance, fermentations were carried out under both configurations, with continuous operation conducted in upward and downward modes, as illustrated in Fig. 2. In batch cultures, the PBB was assembled with all fermentation modules at the beginning of the fermentation and remained in the same configuration until the final time of 96 h. This condition was adopted as the reference for comparison with the continuous-mode assays. For a 4-day fermentation, four fermentation modules were used, designated L1, L2, L3, and L4.

In continuous mode, the modules were added individually every 24 h through a sequence of inoculation, packing, and coupling steps to the PBB. During the initial phase, the bed height was gradually increased until the first inserted module reached the specified residence time. From that point onward, steady-state operation was established, characterized by the withdrawal of fermented modules synchronized with the addition of newly inoculated modules. This movement was performed either upward (inlet at the bottom and withdrawal at the top) or downward (inlet at the top and withdrawal at the bottom), depending on the operational strategy being evaluated. This dynamic enabled uninterrupted fermentation and increased overall productivity, and it was maintained until the scheduled process shutdown, when modules were removed without replacement. Time zero (t = 0) was defined as the beginning of air percolation through the PBB. The total continuous operation lasted 11 days, during which 7 fermentation modules were processed.

In the present study, the term “process time” is used to refer to the total elapsed time of the SSF bioprocess, whereas the term “fermentation time” refers to the time during which the added fermentation modules remained inside the bioreactor for microbial growth and production of the target enzymes. Therefore, in batch operations, process time coincides with fermentation time, whereas in continuous operations, process time is necessarily longer than the fermentation time of each individual module.

#### Respirometric analysis

To estimate *M. thermophila* biomass production during fermentations in the PBB, the CO₂ concentration in the bioreactor outlet stream was measured using a Carbocap GMM 220 probe (Vaisala, Finland), operated by a LabVIEW^®^ routine that recorded the data throughout the fermentation. This approach was adopted byRodrigues et al. [10] because direct sampling during the process was not feasible. Biomass formation was therefore monitored indirectly through observation of the gas-phase composition. The cumulative amount of CO₂ produced (C_i_) was calculated using Eq. (1), from the area under the CO₂ concentration versus cultivation time curve [15].

1 $$\:\mathrm{Ci\:=}\frac{\mathrm{F\:x\:}{\mathrm{CO}}_{\mathrm{2}}\mathrm{\:x\:}\left(\text{}\mathrm{t}\right)\text{}}{{\mathrm{V}}_{\mathrm{m}}}\:\mathrm{+\:}{\mathrm{C}}_{\mathrm{i-1}}$$

where $\:{C}_{i}$is the cumulative amount of carbon dioxide (mol) at time $\:t$(with $\:{C}_{i-1}$representing the number of moles accumulated at the previous measurement time); $\:F$is the airflow rate (L·h⁻¹); CO₂ is the volumetric concentration of carbon dioxide measured by the probe (% v/v); $\:{\Delta\:}t$is the time interval (h); and $\:{V}_{m}$is the molar volume of an ideal gas under ambient conditions (25 L·mol⁻¹).

A logistic growth model was fitted to the cumulative CO₂ curve as a function of cultivation time [16]. Despite its mathematical simplicity, the logistic equation can provide an adequate approximation of the complete growth curve in a single equation, including the lag, exponential-growth, and stationary phases [17], with its integrated form given by Eq. (2), assuming the initial condition X = X_0_ at (t = 0).

2 $$\:\mathrm{X\:=}\frac{{\mathrm{X}}_{\mathrm{m}}}{\mathrm{1+}\left(\frac{{\mathrm{X}}_{\mathrm{m}}}{{\mathrm{X}}_{\mathrm{0}}}\mathrm{\:-\:}{\mathrm{exp}}^{\mathrm{-μt}}\right)}$$

where $\:{X}_{m}$is the maximum amount of carbon dioxide (mol); $\:{X}_{0}$is the initial amount of carbon dioxide (mol); $\:\mu\:$is the specific CO₂ production rate constant (h⁻¹); and $\:t$is the cultivation time (h).

Curve fitting was performed using Microcal Origin^®^ 6.0 software (Microcal Software Inc., Northampton, USA), employing the Levenberg-Marquardt algorithm to estimate the model parameters. Model adequacy was assessed based on the R^2^ value (coefficient of determination) of the fitted curves.

### Evaluation of the downstream operation

At the end of the fermentation period, the material contained in each module was subjected to solid-liquid extraction to obtain the crude enzymatic extract by using the PBB structure itself. In parallel with PBB operation, each module containing the fermented material was individually connected to a solid-liquid extraction system. According to the methodology proposed by Oliveira et al. [11], two sequential recovery steps were employed: percolation extraction and stirred-tank extraction.

Percolation extraction was carried out directly in the module containing the freshly fermented material by replacing the air flow with a distilled-water flow to leach the secreted enzymes. To ensure system operability and avoid cavitation, a fixed solvent volume of 3.1 L per module was used and recirculated in a closed loop by means of a centrifugal pump. This arrangement enabled temperature control and reduced water consumption. Two extraction flow rates, 180 and 2400 L.h⁻¹, were evaluated over 50 min, representing low- and high-flow-rate conditions. To prevent solid carryover, screens with 1 mm openings were installed at the module inlet and outlet.

Subsequently, the material remaining in the bed was subjected to extraction in a stirred tank, which was used as a control to quantify residual enzymatic activity after percolation. This step was performed using distilled water at a ratio of 10 mL.g⁻¹ dry solid (g.d.s.), taking the moisture content of the matrix into account. At the end of each extraction, the crude extracts were vacuum-filtered and centrifuged at 4 °C and 10,000 rpm for 15 min. The supernatants were used for endoglucanase activity (CMCase) determination.

#### Sequential solid-liquid extraction by percolation and evaluation of enzyme recovery efficiency

To further assess endoglucanase recovery, sequential solid-liquid extraction by percolation was performed. This step aimed to quantify cumulative enzyme recovery and to investigate possible limitations associated with liquid flow through a porous lignocellulosic bed.

Unlike the single-flow-rate assays described above, each extraction cycle employed a fresh volume of solvent while maintaining a constant solid-to-liquid ratio. Since each module contained 130 g of dry solid, the solvent volume was rigorously adjusted in each cycle by compensating for the residual moisture of the matrix to ensure an effective and consistent solid-to-liquid ratio.

The contact time was fixed at 50 min per cycle, with upward liquid flow through the bed. At the end of each step, the extract was collected, and the residual solid was immediately subjected to the subsequent cycle under the same operating conditions, totaling three sequential cycles, all performed in triplicate. The enzymatic activity of each fraction was determined individually, and the percentage contribution of each cycle to the total recovery was calculated according to Eq. (3).

3 $$\:\mathrm{Cycle\:contribution\:(\%)=}\frac{{\mathrm{A}}_{\mathrm{j}}}{\sum\:_{\mathrm{j=1}}^{\mathrm{3}}{\mathrm{A}}_{\mathrm{j}}}\times\:100$$

where A_j_ represents the enzymatic activity recovered in cycle j.

### Precipitation

The crude enzymatic extract obtained from the PBB recovery process (10 mL) had its pH adjusted to 4.0 using HCl solutions. Precipitation assays were carried out in triplicate using Falcon tubes, with the total volume varying according to the ammonium sulfate or ethanol concentrations. Precipitation efficiency was evaluated by enzyme recovery (REC%), calculated according to Eq. (4).

4 $$\:REC\:(\% ) = \frac{{A_{{prec \times V_{{prec}} }} }}{{A_{{i\: \times V_{i} }} }} \times 100$$

where $\:{A}_{\mathrm{prec}}$and $\:{A}_{i}$are the enzymatic activities (U·mL⁻¹) of the precipitated and initial fractions, respectively; and $\:{V}_{\mathrm{prec}}$and $\:{V}_{i}$are the corresponding volumes (mL) of the precipitated and initial fractions.

#### Ammonium sulfate precipitation

Salting-out precipitation was performed by varying the final extract saturation from 40 to 90% [18]. The mass of ammonium sulfate (Sigma-Aldrich, USA) required for each saturation range was calculated based on its solubility at 0 °C. The salt was gradually added to the extract under constant magnetic stirring in an ice bath. After complete salt dissolution, the samples were allowed to stand for 6 h at 0 °C, followed by centrifugation at 12,000 × g for 15 min at 4 °C. The precipitate obtained at each saturation level was resuspended in 4 mL of sodium acetate buffer (0.1 M, pH 4.0) for subsequent determination of enzymatic activity and recovery efficiency.

#### Ethanol precipitation

For ethanol precipitation, final concentrations ranging from 60 to 90% (v/v) were evaluated [19]. Absolute ethanol (Sigma-Aldrich, USA), previously cooled to -20 °C, was added dropwise to the enzymatic extract (10 mL) maintained in an ice bath. After homogenization, the mixtures were incubated at 0 °C for 6 h. The precipitated fractions were separated by centrifugation (12,000 × g, 15 min, 4 °C), resuspended in 4 mL of sodium acetate buffer (0.1 M, pH 4.0), and stored at 0 °C for enzymatic activity quantification.

#### Precipitation kinetics

The effect of equilibrium time on precipitate formation was evaluated under the two saturation conditions that showed the best results in the previous step. The precipitating agent was added to the extract (pH 4.0) under constant stirring at 0 °C. Aliquots were collected at predetermined intervals (0, 1, 2, 4, 6, 12, and 24 h) and immediately centrifuged (12,000 × g, 15 min, 4 °C). The sediment was recovered and solubilized in buffer to determine the minimum incubation time required for maximum endoglucanase recovery.

### Evaluation of enzyme activity at different temperatures and pH values

To determine the optimal pH and temperature conditions for endoglucanase activity, enzymatic assays were performed by simultaneously varying these two variables over a pH range of 4.0, 5.0, and 6.0 and at temperatures of 50, 55, and 60 °C. The experiments were conducted using the precipitated extract. The effect of pH and temperature was evaluated using 0.1 mol.L⁻¹ sodium acetate buffer containing 2% (w/v) carboxymethyl cellulose (CMC) as substrate. Sodium acetate buffer solutions at 0.2 mol L⁻¹ were adjusted to pH 4.0, 5.0, and 6.0 and then diluted to the working concentration. The enzymatic reactions were carried out at the indicated temperatures with an incubation time of 10 min.

To minimize pH variations resulting from the temperature dependence of pKa, the buffer pH values were corrected based on tabulated data from the literature [20]. The optimal conditions were defined as those yielding the highest endoglucanase activity.

### Thermostability

The thermal stability of the precipitated endoglucanase was evaluated using a methodology adapted from Zhou et al. [21]. Aliquots of 5 mL of crude and precipitated enzymatic extract were incubated for 24 h after 1:1 (v/v) resuspension in 0.05 mol L⁻¹ sodium acetate buffer adjusted to the optimal pH and temperature determined in Sect.  2.5. Samples were collected at initial 10 min intervals and subsequently at 1, 2, 4, 6, 12, and 24 h, and were immediately cooled in an ice bath to stop inactivation. The thermostability data were fitted to the kinetic inactivation model proposed by and Sadana et al. [22], using Eq. (5). The half-life (t _1/2_) was calculated using Eq. (6).

5 $$\frac{A}{{A_{0} }} = \left( {1 - \alpha } \right) \times ~e^{{ - k \times t}} + \alpha$$

6 $$t_{{1/2}} = - \frac{1}{k}\ln \left( {\frac{{0.5 - \alpha }}{{1 - \alpha }}} \right)$$

where A/A_0_ is the dimensionless relative activity; $\:\alpha\:$is the ratio between the specific activities of the initial and final states; $\:k$is the first-order deactivation rate constant (h⁻¹); and $\:t$is the incubation time (h).

### Storage analysis

Aliquots of precipitated endoglucanase extract were stored for 180 days to identify the most suitable condition for preserving enzymatic activity. The evaluated conditions included storage in a refrigerator (5 °C), a conventional freezer (-20 °C), at room temperature (24 °C), and in an ultra-freezer (-80 °C), as well as a condition with the addition of glycerol (20% v/v) as a cryoprotectant for ultra-freezer storage.

Residual endoglucanase activity was evaluated every 30 days under the optimal pH and temperature conditions. Frozen samples (-20 °C and − 80 °C) were thawed by immersion in a water bath at 25 °C immediately before analysis. The results were expressed as relative activity (%), considering the activity determined immediately after extraction as 100%. All assays were performed in triplicate for each condition.

### Analytical determinations

#### CMCase activity of the extracts

Endoglucanase (CMCase) activity was determined according to Ghose [23], using 2% (w/v) crboxymethyl cellulose (CMC, Sigma-Aldrich) in 0.1 mol.L⁻¹ sodium acetate buffer (pH 5.0). The released reducing sugars were quantified by the 3,5-dinitrosalicylic acid method Miller [24]. Absorbance was measured at 540 nm using a UV–Vis spectrophotometer. One enzymatic unit (U) was defined as the amount of enzyme capable of releasing 1.0 µmol of glucose per minute of reaction per mL of enzyme. Activities were expressed as U.mL⁻¹ and converted to units per gram of dry solid substrate (U·g.d.s⁻¹) based on the extraction-volume ratio (mL·g.d.s⁻¹).

#### Final moisture content

The final moisture content of the fermented solids was determined using an M5 Thermo infrared moisture analyzer (Bel Engineering, Monza, Italy). Samples of 2 g were dried at 110 °C until constant moisture content (± 0.1%) was reached. Sampling was carried out randomly from each module of the packed-bed bioreactor.

#### Statistical analysis

All analyses were performed in triplicate, and reactor experiments were carried out in duplicate. Data are presented as mean ± standard deviation. Minitab^®^16 software (Minitab Inc., State College, USA) was used to perform analysis of variance (ANOVA) and Tukey’s test for comparison of means, adopting a 95% confidence level.

## Results and discussion

### Solid-state fermentations in packed-bed bioreactor

#### Thermal behavior during fermentation

**Fig. 3:**
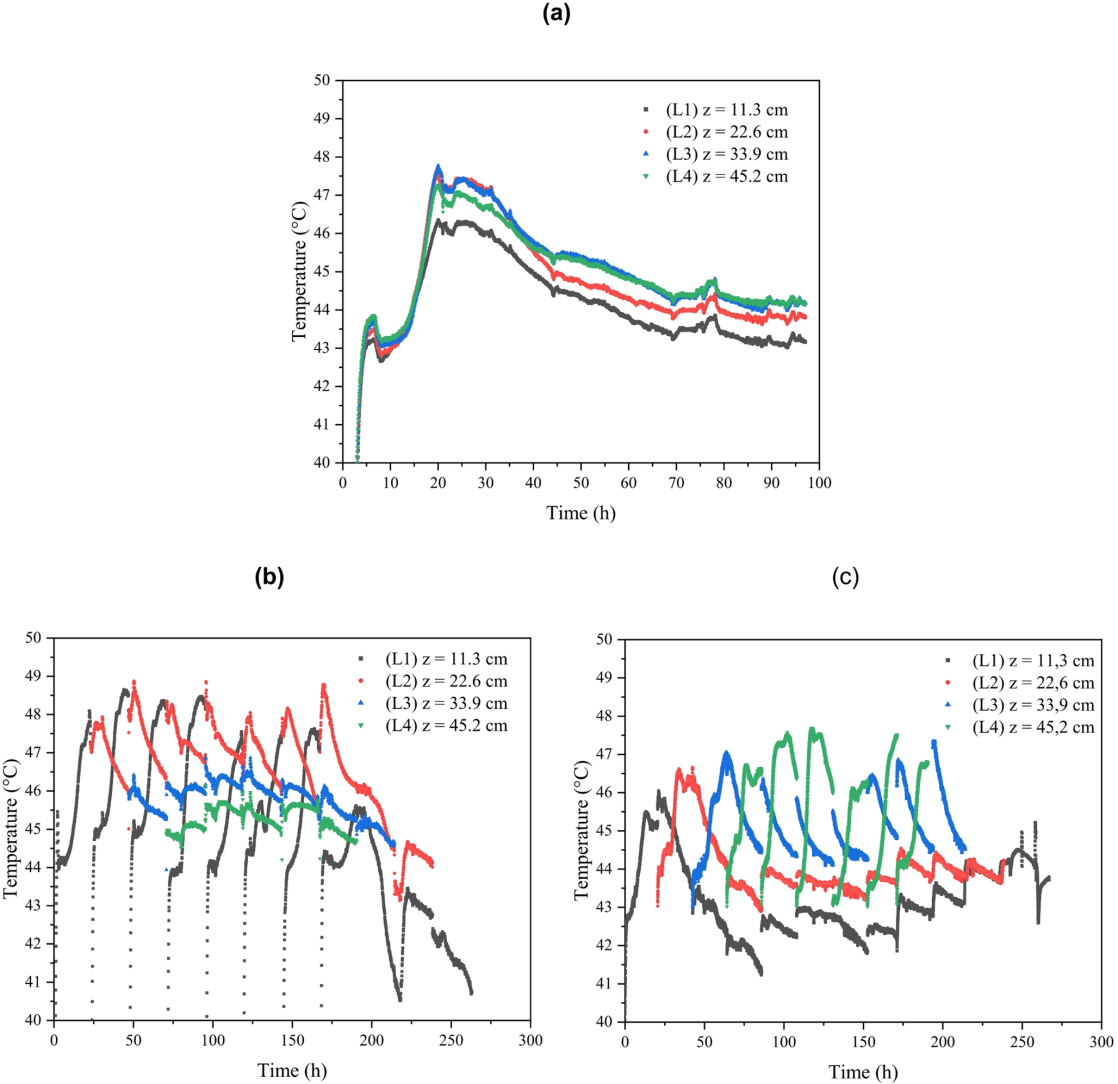
Temperature profiles of solid-state fermentations of *M. thermophila* I-1D3b in a packed-bed bioreactor (PBB) using SCB: WB 7:3 (w/w) substrate under (**a**) batch, **b** upward continuous, and **c** downward continuous operating modes

Thermal regulation remains one of the main technical challenges in the operation of packed-bed bioreactors, since the accumulation of metabolic heat may compromise enzyme stability and the homogeneity of the fermentation bioprocess [9]. In this context, the influence of batch and continuous operating regimes on the thermal behavior of the bed during endoglucanase production by *Myceliophthora thermophila* I-1D3b on a lignocellulosic substrate (SCB: WB, 7:3 w/w) was investigated. Figure 3 shows the temporal evolution of the temperatures monitored in the four fermentation modules for batch operation (Fig. 3a), upward continuous operation (Fig. 3b), and downward continuous operation (Fig. 3c).

As shown in Fig. 3, the average temperatures recorded throughout the fermentations remained close to 45 °C, with variations of less than 4 °C among the monitored points. Under batch operation, an initial temperature increase was observed, followed by a thermal peak and subsequent stabilization or slight decrease, a behavior consistent with the transient accumulation of metabolic heat in fixed-bed systems [8]. The formation of axial thermal gradients is intrinsically associated with unidirectional aeration and was evidenced by the dispersion of temperatures along the bioreactor length. Although overheating was slight, the persistence of gradients may affect oxygen mass transfer and the evaporation rate within the bed [9, 10].

Under continuous operation (Fig. 3b and c), the thermal profile showed more frequent oscillations than in batch mode, reflecting the intermittent movement of fermentation modules throughout the process. Despite these fluctuations, the temperatures remained within a range similar to that observed in batch operation, with no evidence of relevant thermal overheating. Between 96 and 210 h, with the addition and removal of one module every 24 h, the system operated with successive advancement of the modules along the column, resulting in behavior close to that of a plug-flow reactor. This interpretation is consistent with Mitchell et al. [25], who indicated that continuous PBB operation may promote greater uniformity of process conditions along the bed. Maximum and average temperatures were similar among the operating regimes, with peak values of 48.2 ± 0.6 °C (batch), 48.9 ± 0.7 °C (upward continuous), and 47.8 ± 0.8 °C (downward continuous), and average temperatures of 44.7 ± 0.3, 44.5 ± 0.4, and 44.5 ± 0.5 °C, respectively, with no significant differences observed (*p* > 0.05). These results indicate that, under the evaluated conditions, both operating modes maintained the system close to the desired thermal range, without severe overheating.

The maintenance of the temperature within a range close to the optimum suggests that the combination of high bed porosity, use of a thermophilic microorganism, and the adopted module-movement strategy favored dissipation of metabolic heat. In the present study, bed porosity was 0.764, which is higher than the value reported by Perez et al. [26] for a system containing sugarcane bagasse and wheat bran, in which a temperature rise of approximately 3 °C was observed at a porosity of 0.55. In contrast, Ghildyal et al. [27] reported a temperature increase of 15 °C during cultivation of *Aspergillus niger* on wheat bran in a PBB. Taken together, these comparisons reinforce that the physical structure of the bed plays a decisive role in heat dissipation and in the intensity of thermal gradients in solid-state fermentation.

In addition to the operating mode, the position at which new modules were introduced also influenced the reactor thermal profile. Figure 3c shows the thermal behavior of continuous fermentation when freshly inoculated modules were added through the upper part of the reactor. In this configuration, temperature peaks were concentrated in the upper regions of the PBB, in contrast to the profile observed in Fig. 3b, in which the highest peaks occurred in the lower and intermediate regions of the bed. This difference indicates that the position occupied by newly inoculated modules directly influences the spatial distribution of heat within the system.

In Fig. 3c, the temperature drops recorded every 24 h reflect the moments of module addition, removal, and movement, characterizing the intermittent regime of continuous operation. Since the new modules were inserted at the upper position of the fermentation zone, thermal peaks occurred preferentially at higher reactor heights, whereas positions closer to the air inlet showed smaller thermal variations over time. This behavior indicates that the module-feeding strategy alters the longitudinal heat distribution in the PBB and should therefore be considered in the operational design of the system. However, due to operational constraints associated with integration with the downstream system, the subsequent continuous experiments in this study were conducted in the upward mode, with module addition through the lower part of the reactor.

#### Enzymatic activity and moisture

**Fig. 4:**
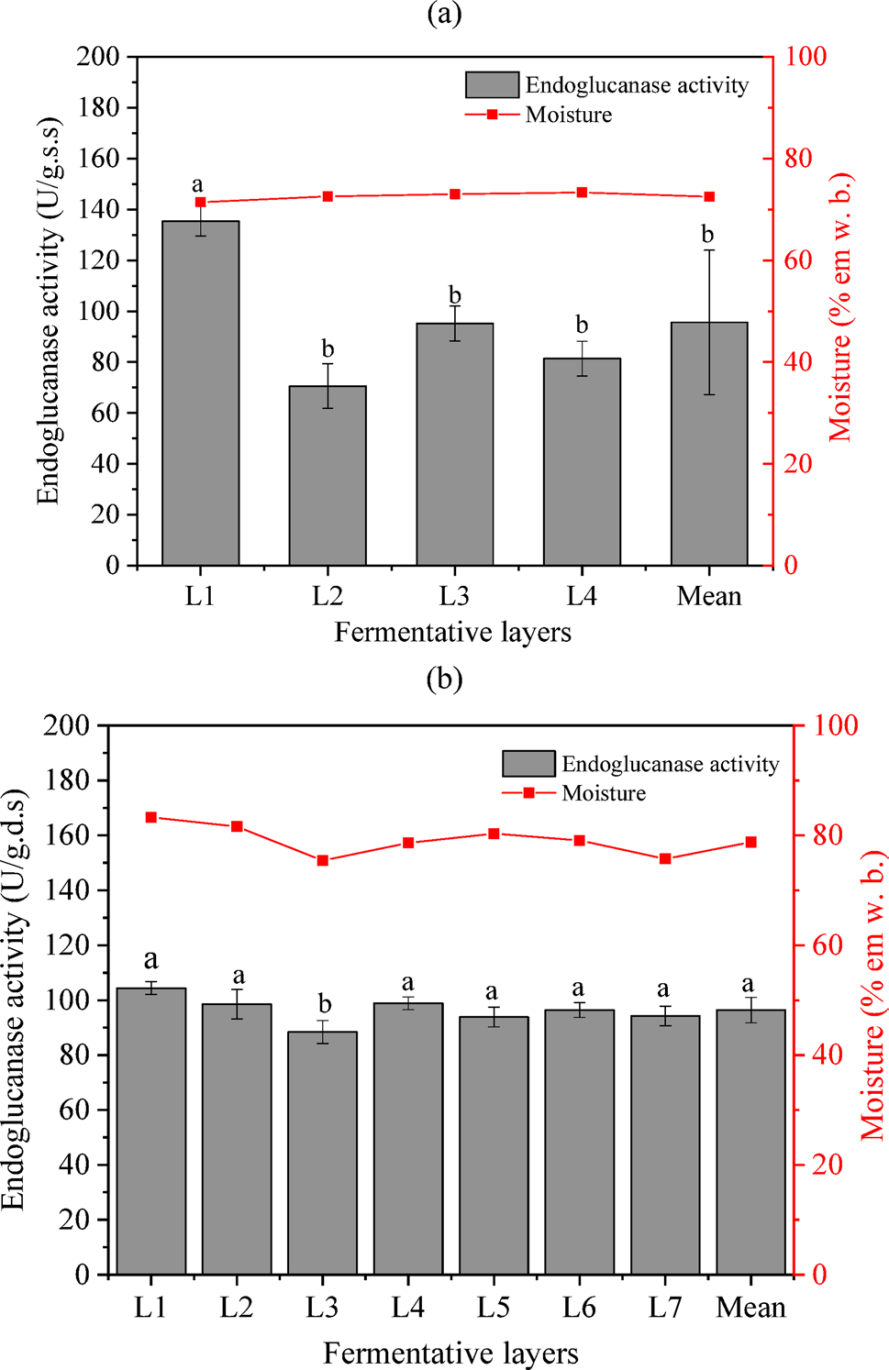
Endoglucanase activity and final moisture content of fermentations in a packed-bed bioreactor with 4 and 7 fermentation modules using SCB: WB 7:3 substrate and *M. thermophila* under (**a**) batch and (**b**) continuous operation. Subscript L indicates the layers

Endoglucanase activity and the final moisture content of the fermentation modules were evaluated under batch and upward continuous operation, as shown in Fig. 4a and b and on Table 1. Control of these variables is critical for PBB performance, since hydrothermal gradients lower than 10% may reduce enzyme productivity by 30–50% [28]. The average endoglucanase activity was similar for batch and continuous operation, with values of 95.63 ± 28.37 and 96.36 ± 4.63 U·g.d.s^− 1^ respectively. Despite the similarity between the mean values, the lower dispersion observed in the continuous process indicates greater spatial homogeneity of enzymatic activity among the fermentation modules. Thus, the main difference between the operating modes was associated with the spatial uniformity of the process rather than with the average level of enzyme production.

This homogeneity under continuous operation contrasts with the greater dispersion observed in batch mode, which can be attributed to local heterogeneities in moisture, temperature, and oxygen availability that are characteristic of static systems, and it represents greater process robustness and predictability from an operational standpoint. This higher uniformity is associated with the progressive movement of the modules along the bioreactor, causing all of them to be exposed over time to all regions of the thermal and moisture gradients. According to Mitchell et al. [25], under continuous operation the PBB tends to approach plug-flow reactor behavior, favoring more uniform exposure of the layers to the gas-phase and thermal conditions within the system.

These results are consistent with those reported by Rodrigues et al. [10], who also observed greater homogeneity of endoglucanase activity in continuous fermentations with *M. thermophila* I-1D3b in a PBB using an SCB: WB 7:3 (w/w) substrate.

In addition to enzymatic activity, bed moisture was also a critical aspect of the system, since a reduction in water content directly impairs fungal growth and metabolic activity in SSF [28]. The final moisture contents of the solid matrix, expressed on a wet basis (% w.b.), are shown in Table 1.

**Table 1:** Final moisture content of the inlet and outlet accessory modules and fermentation modules of the packed-bed bioreactor after solid-state fermentation with *M. thermophila* using sugarcane bagasse and wheat bran (7:3, w/w) under batch and continuous operation

Operating mode	Inlet module (% w.b.)	Fermentation modules (% w.b.)	Outlet module (% w.b.)
Batch	33.35 ± 1.70^a^	72.49 ± 1.95^a^	12.12 ± 1.78^a^
Continuous	13.49 ± 2.70^b^	78.78 ± 2.72^b^	38.13 ± 2.41^b^

*For each column, means that do not share a letter are significantly different according to Tukey’s test (*p* < 0.05)

The final moisture contents in the fermentation modules were 72.49 ± 1.95% w.b. for batch operation and 78.78 ± 2.72% w.b. for continuous operation, both values being close to the initial condition of 75% w.b. In continuous operation, the slightly higher value suggests that substrate movement combined with the humidification system limited water losses along the bed. Under batch operation, the profile indicates localized drying in the inlet region, as expected in static systems under continuous airflow. The data in Table 1 also highlight the role of the accessory modules as moisture-buffering zones. In the continuous process, the longer operating time intensified the gradient between the end modules, with more pronounced drying at the inlet and water accumulation at the outlet, confirming that these modules protected the fermentation bed from system-related moisture variations. Periodic replacement of their contents every 96 h proved to be an effective strategy for preserving bed stability.

The combined analysis of Fig. 4; Table 1 indicates that the moisture variations among the modules were not sufficient to compromise endoglucanase production. Although module 1 showed greater water loss under batch operation, no corresponding reduction in enzymatic activity was observed, suggesting that the maintained moisture range remained compatible with the metabolism of *M. thermophila* I-1D3b. These findings are supported byCasciatori et al. [9] and Katayama et al. [29], who demonstrated that accessory modules act as effective barriers against water loss and excessive bed saturation. In contrast, Perez et al. [30] reported severe drying and flooding in the absence of these modules, reinforcing their importance for moisture control in the system.

#### Respirometric analysis

**Fig. 5:**
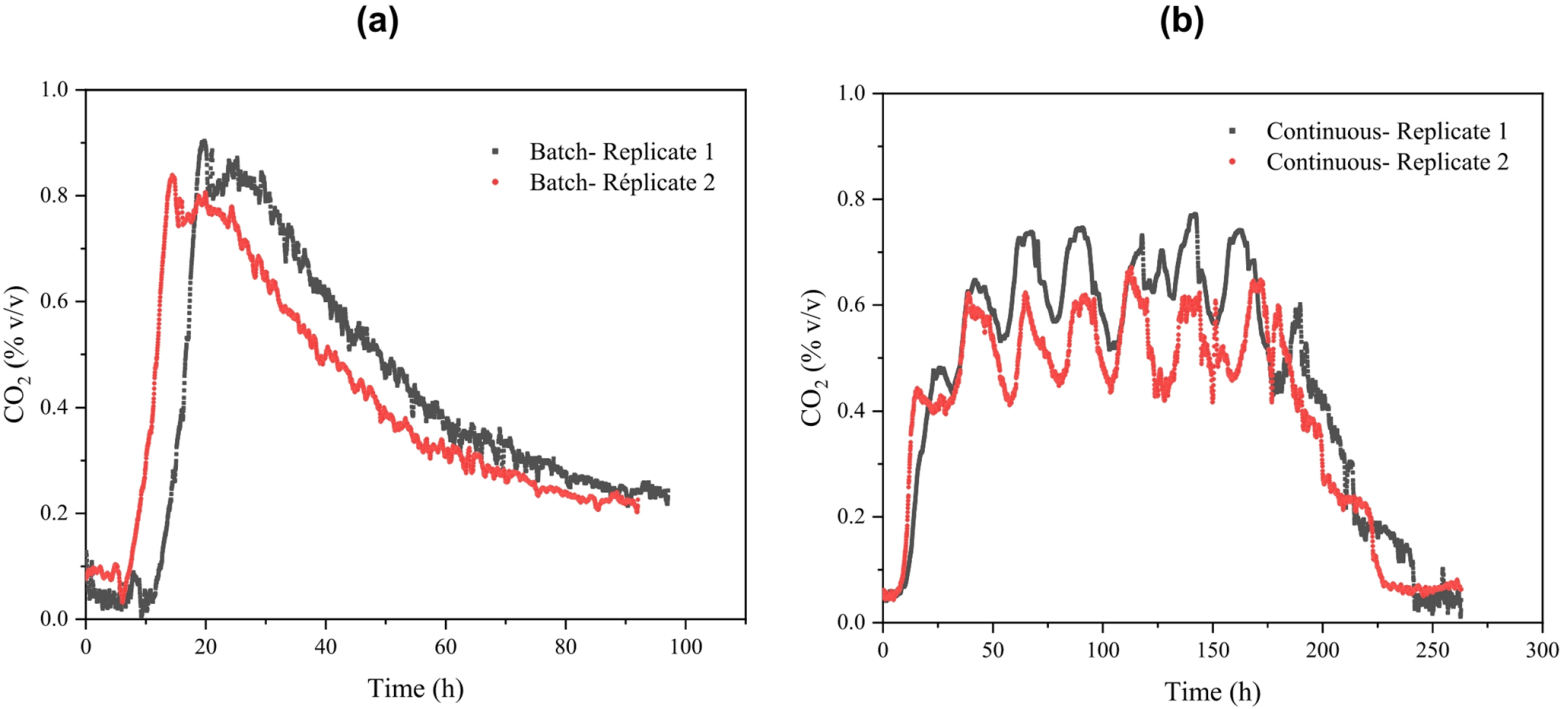
Duplicate CO₂ concentration profiles in the outlet gas stream of the PBB for cultures operated in (**a**) batch mode and (**b**) continuous mode

Direct biomass determination in SSF is limited because fungal hyphae penetrate the substrate particles, making it impossible to separate out the biomass from the solid matrix [31]. Monitoring the CO₂ concentration in the bioreactor outlet air therefore constitutes an indirect indicator of metabolic activity throughout fermentation. Figure 5 presents, in duplicate, the profiles of volumetric CO₂ concentration (% v/v) for batch operation (Fig. 5a) and upward continuous operation (Fig. 5b), obtained with four fermentation modules in operation. These profiles showed the same behavior observed for the temperature profiles, with peaks between 17 and 24 h of fermentation, confirming that the recorded thermal variations were indeed caused by metabolic heat generation, as also reported by Rodrigues et al. [10].

In batch mode, as shown in Fig. 5a, the CO₂ concentration increased rapidly, reaching a peak associated with the exponential phase of microbial growth, followed by a gradual decline, indicating a reduction in metabolic activity due to nutrient depletion and accumulation of inhibitory metabolites. This behavior is typical of closed systems and has been widely described in the literature [8].

In continuous mode, as shown in Fig. 5b, CO₂ emission occurred more uniformly over time, without a pronounced peak. This pattern is associated with the greater operational stability of the system, in which the fermentation modules are at different growth stages; while some modules are in the logarithmic phase, others are in the lag, deceleration, or stationary phase, which reduces the overall metabolic intensity and, consequently, the maximum CO₂ concentration relative to batch operation. This behavior was reflected in the maximum recorded CO₂ values, namely 0.904% (v/v) in batch mode and 0.772% (v/v) in continuous mode, corresponding to a difference of approximately 15%, in agreement with the simulations of Mitchell et al. [25], according to which the metabolic heat generation rate under continuous steady-state operation corresponds to about 57% of the maximum value predicted for batch operation.

From the cumulative CO₂ data, specific growth constants (µ) were estimated by fitting kinetic models. The logistic equation provided the best fit for both operating modes, with high coefficients of determination (R²), similar to those reported by Rodrigues et al. [10], who obtained values between 0.97 and 0.98. The µ value estimated for continuous operation was 0.017 ± 0.001 h⁻¹, significantly lower than that obtained for batch operation, which was expected since, in continuous mode, the modules operate simultaneously at different growth stages, resulting in lower average values. The values obtained for batch operation, 0.080 ± 0.001 h⁻¹, are consistent with those reported by Casciatori et al. [17], a µ value of 0.06 h⁻¹ for *M. thermophila* I-1D3b, and by Henrique et al. [32], who reported values close to 0.07 h⁻¹ for the same species.

Considering only the steady-state period of continuous operation, between 96 and 192 h, when the system operated with four fermentation modules, greater cumulative CO₂ production was observed than in batch mode due to the longer process time relative to the batch runs. A linear relationship between cumulative CO₂ production and time was observed in this interval, indicating plug-flow behavior of the PBB, in which each module contributes additively and sequentially to the total CO₂ production and accumulation, in agreement with [8].

### Downstream processing

Downstream processing, particularly enzyme extraction, concentration, and stabilization, represents a significant fraction of the total cost of cellulase production by solid-state fermentation (SSF) and remains a bottleneck for industrial implementation [33]. In this context, any process intensification in this sense is relevant. The approach used in the current work was to take each spent layer from the continuous PBB directly to a parallel column so that endoglucanase recovery could be evaluated via solid–liquid extraction by ascendant distilled-water percolation in packed-bed in a closed loop. Two extraction flow rates (180 and 2400 L.h⁻¹, applied for 50 min) were compared with conventional stirred-tank extraction.

Although the volume of each module is approximately 1.3 L, the relatively high flow rates were intentionally selected because cellulolytic enzymes are known to adsorb strongly onto the lignin fraction of lignocellulosic substrates, which can hinder their recovery [34]. Under these conditions, high percolation rates increase liquid-solid contact and reduce external mass transfer limitations, promoting enzyme desorption and transport from the solid matrix into the extraction liquid. Consequently, the enhanced recovery obtained at higher flow rates is likely associated with improved leaching efficiency rather than simply the volume of solvent employed.

Regarding the energy requirements, high recirculation rates may increase pumping costs. However, in the context of integrated lignocellulosic biorefineries, a significant fraction of the energy demand can be supplied through cogeneration systems based on residual biomass streams. Nevertheless, a detailed techno-economic assessment would be necessary to determine the optimal balance between enzyme recovery efficiency and operational energy consumption at larger scales.

**Fig. 6:**
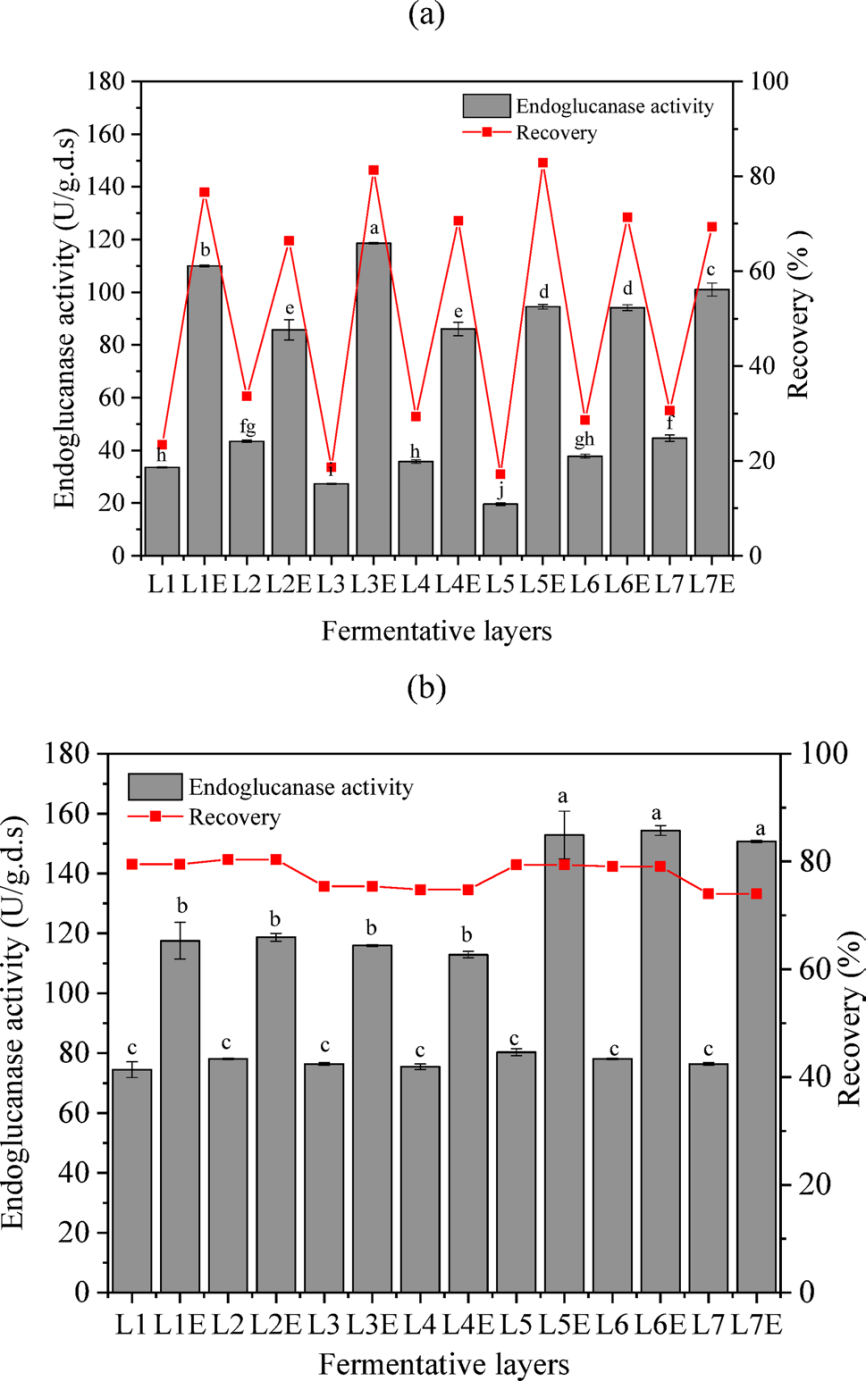
Endoglucanase activity and recovery per module obtained from SSF assays with *M. thermophila* (SCB: WB ratio of 7:3 w/w) in a packed-bed bioreactor under continuous operation. Extraction flow rates were (**a**) 180 L h⁻¹ and (**b**) 2400 L h⁻¹. The subscript “E” indicates the activity recovered by percolation, while values without a subscript represent the residual activity from the complementary extraction of the percolated solid

Figures 6a and b present CMCase activity recovered from the seven fermentation modules for the evaluated flow rates 180 and 2400 L.h⁻¹, respectively, distinguishing the activity extracted by percolation (subscript “E”) from the residual activity obtained by subsequent conventional extraction of the percolated solids in stirred-tank mode. Each data point corresponds to the average of two independent continuous fermentations for each condition. No bed compaction or preferential channeling was observed, indicating adequate hydrodynamic behavior and uniform liquid distribution within the porous matrix.

At 180 L·h⁻¹ (Fig. 6a), greater variability in enzymatic activity among modules was observed, which may be attributed to differences in percolation fluid residence time and local operating conditions affecting enzyme extraction. Anyhow, under this condition, percolation extraction resulted in higher activities than those obtained by single-stage stirred-tank extraction (96.36 ± 4.63 U·g.d.s⁻¹), indicating that in situ and dynamic recovery within the packed bed enhances enzyme removal efficiency, even at the lower water flow rate tested. This behavior may be attributed to the continuous flow of the extraction liquid through the packed bed, which enhances convective mass transfer and facilitates the desorption of enzymes bound to the lignocellulosic matrix [35]. As a result, local mass transfer limitations are reduced, leading to more efficient enzyme recovery than that achieved by single-stage stirred-tank extraction.

At 2400 L·h⁻¹ (Fig. 6b), enzymatic activity showed higher average values, reaching 131.86 ± 19.56 U·g.d.s⁻¹, whereas the residual activity was 77.03 ± 1.95 U·g.d.s⁻¹. In comparison, at 180 L·h⁻¹ (Fig. 6a), greater variability in enzymatic activity among modules was observed, likely due to differences in percolation fluid residence time and local operating conditions affecting enzyme extraction; under these conditions, percolation resulted in activity of 98.57 ± 12.21 U·g.d.s⁻¹, with residual activity of 34.56 ± 8.85 U·g.d.s⁻¹. Tukey’s test indicated significant differences both between flow rates and between extraction modes, demonstrating that the percolation flow rate directly affects recovery efficiency. The 2400 L·h⁻¹ condition maximized the total extracted activity, although a still substantial enzymatic fraction remained associated with the solid support, suggesting that extraction was enhanced but not exhaustive.

These results are consistent with percolation strategies reported in the literature, in which increasing the flow rate or flow intensity significantly enhanced recovery of soluble proteins and endoglucanase, reaching up to 57% recovery of total proteins and 88% specific recovery of endoglucanase [11]. Such gains have been associated with intensified mass transfer between the lignocellulosic matrix and the extracting liquid, especially in systems in which the enzymes interact strongly with the substrate [34, 35], as aforementioned.

From a process-engineering perspective, percolation extraction coupled to the packed-bed bioreactor presents operational advantages over the conventional method, since it allows integration of the enzyme-recovery step directly with SSF, without the need for handling or transferring the cultivated solid, and with potential reduction in the volume of extracts generated. Even so, part of the activity remains retained in the biomass even at high flow rate, in agreement with studies highlighting the role of lignin as an adsorption site for cellulases, thereby reducing the fraction recoverable by simple aqueous washing [36]. This reinforces the attractiveness of routes based on the direct use of the fermented material as a solid biocatalyst in subsequent biorefinery steps, particularly in saccharification and second-generation ethanol production processes [37].

#### Solid-liquid extraction cycles

**Fig. 7:**
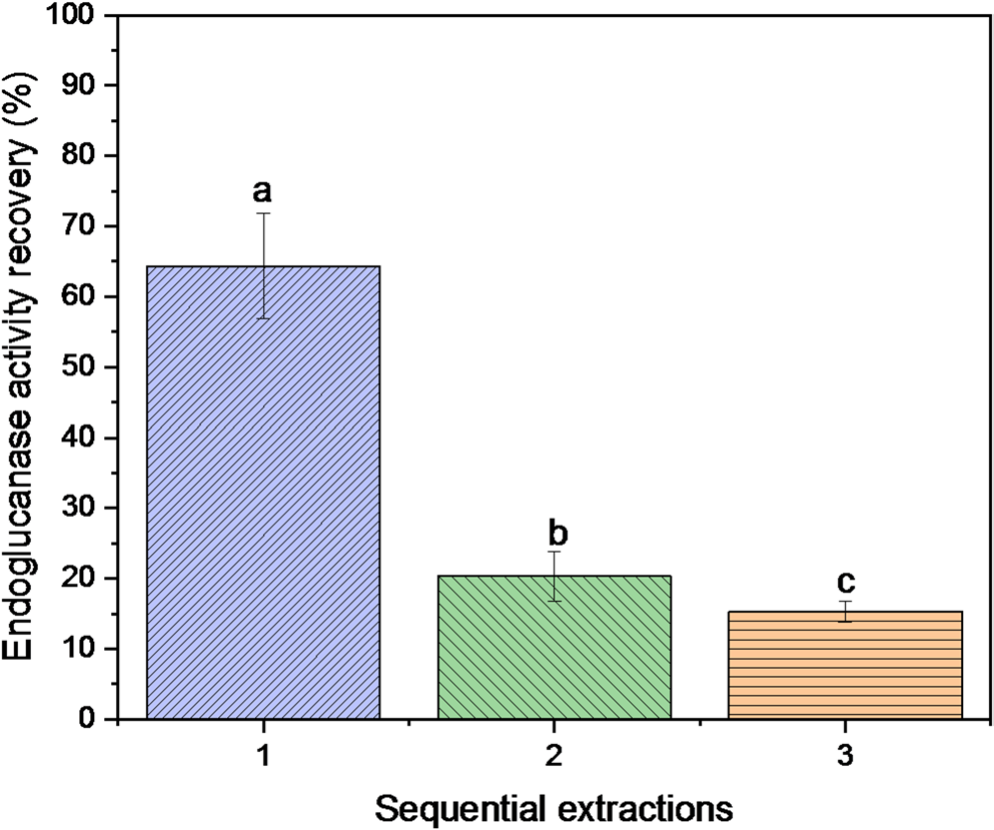
Endoglucanase activity recovered per sequential percolation extraction cycle from *Myceliophthora thermophila* I-1D3b fermented substrate (sugarcane bagasse: wheat bran, 7:3 w/w). Bars represent mean ± standard deviation (*n* = 3). Different letters indicate significant differences by Tukey’s test (*p* < 0.05)

The efficiency of enzyme recovery is a critical aspect of the feasibility of SSF processes, since the extraction step directly influences both the available activity and the downstream load. Solid-liquid extraction with water, whose results are shown in Fig. 7, recovered only part of the endoglucanase activity produced by *M. thermophila* I-1D3b. The first extraction cycle, carried out for 50 min, recovered 64% of the total leachable activity, considering the sum of the three cycles, whereas the second and third cycles contributed only 20% and 16%, respectively, demonstrating diminishing returns as the number of extractions increased.

This behavior is consistent with previous reports on recovery of cellulases produced by SSF, in which the largest enzyme fraction is removed in the first cycle and additional extractions generate progressively more diluted extracts, producing marginal gains at the expense of higher water consumption and greater processing complexity [36].Catalán et al. [38] andMarín et al. [33] reported 75% recovery in the first aqueous extraction of cellulases and approximately 25% in subsequent extractions, a pattern similar to that observed in the present study. In lignocellulosic matrices, this pattern has been associated with heterogeneous enzyme distribution in the substrate and partial retention of activity in the residual biomass, with the efficiency of successive steps depending on the type of enzyme and the characteristics of its interaction with the solid matrix [39].

The incomplete recovery observed even after three 50 min cycles is consistent with the hypothesis of enzyme retention in the solid matrix, especially in lignin-rich substrates. The non-productive adsorption of cellulases onto lignin has been identified as an important factor reducing the enzymatic fraction available in solution and, consequently, limiting the efficiency of simple aqueous extraction [35]. Since this mechanism was not directly evaluated in the present study, this interpretation should be regarded as plausible rather than conclusive.

Additional extraction cycles increased not only the water demand, but also the total operating time and downstream load due to the generation of larger extract volumes. The results therefore indicate that the definition of the number of cycles should balance recovered activity, process time, water consumption, and operational complexity.

### Endoglucanase precipitation with ammonium sulfate and ethanol

**Fig. 8:**
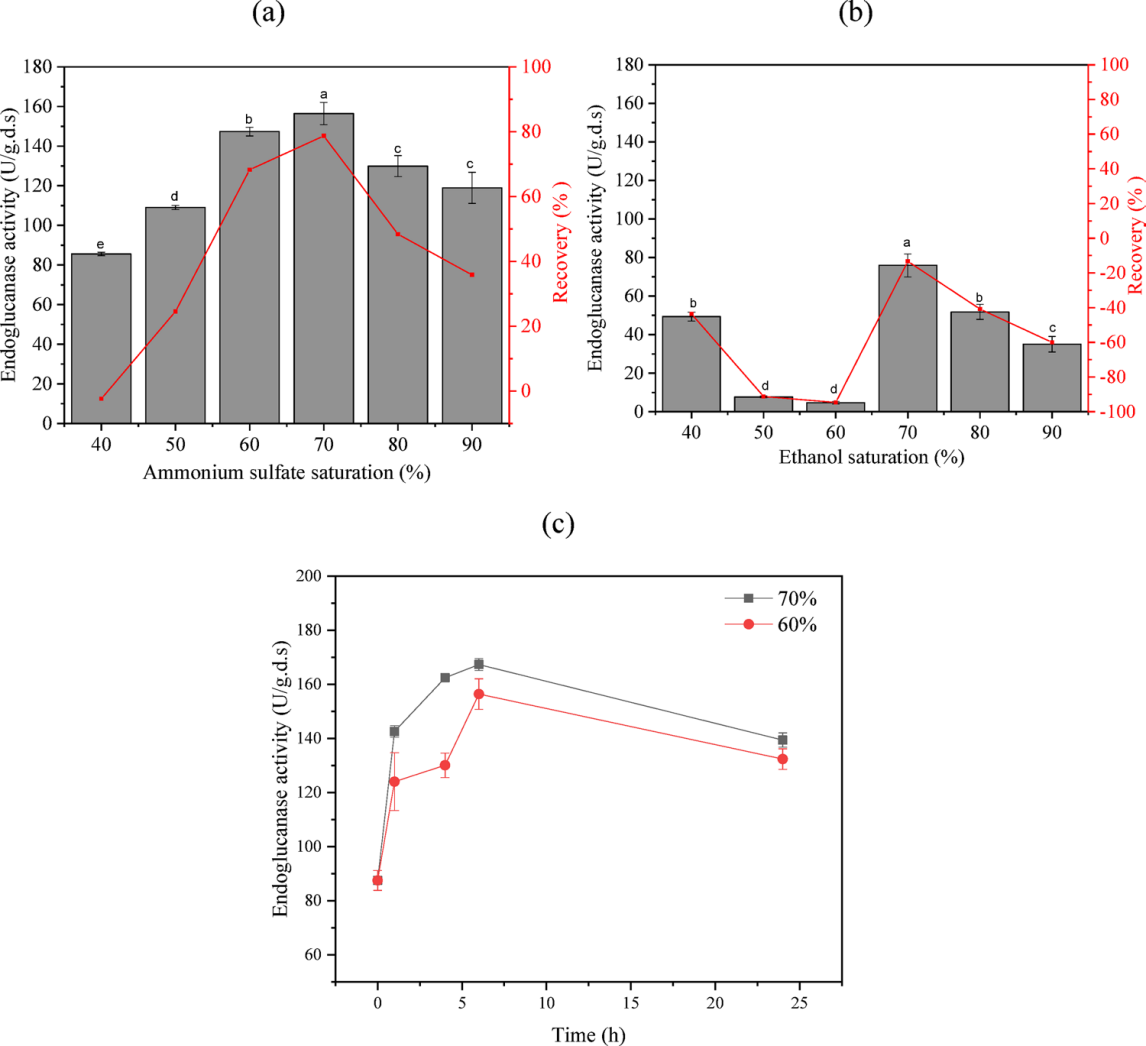
Endoglucanase activity as a function of (**a**) ammonium sulfate saturation, **b** ethanol concentration, and **c** kinetics of endoglucanase precipitation with ammonium sulfate at 60 and 70% saturation

Precipitation is a protein concentration and fractionation step whose performance depends on the type of precipitating agent and its concentration [40]. Figure 8 presents the results obtained using ammonium sulfate (Fig. 8a) and ethanol (Fig. 8b) as precipitating agents for endoglucanase from *M. thermophila* I-1D3b, as well as kinetics of precipitation (Fig. 8c).

In ammonium sulfate precipitation, activity increased progressively up to 70% saturation, at which the highest activity and best enzyme recovery were obtained, reaching 156.44 U·g.d.s^− 1^ and a recovery yield of 78.69% relative to the crude extract. A saturation of 60% also resulted in substantial recovery, with 147.34 U·g.d.s^− 1^ and an yield of 68.29%, and both conditions were selected for the subsequent kinetic study. Residual activity followed a similar trend within this range, indicating that the enzyme maintained its structural integrity within the optimal saturation interval. Above 70%, both activity and recovery decreased, indicating that excess salt impaired protein conformation. In the case of ethanol, none of the evaluated concentrations exceeded the activity of the crude extract, with negative recovery values under all tested conditions and a maximum activity of 75.91 U·g.d.s^− 1^, observed at 70%, indicating that this agent caused a net loss of enzymatic activity in the studied system.

These results indicate that ammonium sulfate promoted a more selective fractionation of endoglucanase, with better retention of catalytic activity after precipitate redissolution. The increase in activity relative to the crude extract suggests that, in addition to enzyme concentration, there was partial removal of extract components that interfered with the assay. The precipitation mechanism involves an increase in ionic strength caused by salt, which reduces electrostatic repulsion between protein molecules and strengthens hydrophobic interactions, thereby favoring aggregation without compromising the native conformation of the enzyme [41].

In the case of ethanol, the negative recovery values indicate that the organic solvent, under the tested conditions, not only failed to concentrate the enzyme but also caused activity loss relative to the crude extract. This behavior is associated with the ability of ethanol to dehydrate protein molecules, promoting disruption of hydrophobic interactions and exposure of nonpolar groups, which compromises the native enzyme conformation at higher concentrations [42]. Mariño et al. [43] observed that cellulase recovery by ethanol precipitation from a *Trichoderma harzianum* fermented broth depended critically on specific pH, temperature, and solvent-concentration conditions, requiring pH 5.0 and 5 °C to achieve satisfactory recoveries. These conditions were not employed in the present study, which may explain the poorer performance of ethanol. The comparison between initial and residual activity confirms that ethanol precipitation had a more detrimental effect on enzyme stability than ammonium sulfate.

Qin et al. [44] purified a thermostable endoglucanase from *Aspergillus oryzae* by ammonium sulfate precipitation followed by anion-exchange chromatography, showing that this salt is an effective pre-purification strategy for this enzyme class.Nisar et al. [45] used ammonium sulfate precipitation as the first purification step for cellulases from *Thermomyces dupontii*, further supporting the applicability of this method to thermoactive fungal enzymes.Han et al. [46] reported that the endoglucanase from *Chaetomium thermophilum* was concentrated in the 80% ammonium sulfate saturation fraction, a value close to that obtained in the present work, and that endoglucanase from *Fomitopsis meliae* fractionated with acetone showed a recovery of 74.33%, indicating that organic solvents may also be used to fractionate this class of enzymes.Farinas et al. [31] obtained endoglucanase recoveries of up to 61% by ethanol precipitation, although under optimized temperature and aging-time conditions, reinforcing that the performance of this precipitating agent is strongly dependent on the adopted operating conditions.

Since 60 and 70% saturation provided the highest enzyme recoveries, the effect of salt-exposure time under these conditions was evaluated, considering that contact time may alter the recovered activity during precipitate formation. Figure 8c shows a rapid initial increase in recovered activity, followed by a tendency toward stabilization at longer times for both saturation levels, indicating that most of the precipitation occurred during the initial stages of the process. This behavior is related to the ability of salt to reduce the solvation layer around the enzyme, promoting intermolecular interactions that facilitate enzymatic aggregation [47, 48]. The lower standard deviations at intermediate times suggest greater reproducibility in this range, whereas the greater dispersion at longer times indicates lower preparation stability.

Koteshwara et al. [48] reported a progressive reduction in enzymatic activity with increasing incubation time in ammonium sulfate, with better performance at 20 min than at 3 h and 8 h, attributing this loss to the possibility of aggregation or denaturation during prolonged salt exposure, an interpretation consistent with the trend observed in Fig. 8c. Farinas et al. [31] also observed that precipitant concentration and exposure time had a statistically significant effect on endoglucanase recovery, reinforcing that both variables should be considered jointly when optimizing the precipitation step.

### Effect of pH and temperature on enzymatic activity

pH and temperature are key determinants of cellulase catalytic activity, as they define the appropriate operating range for applications in lignocellulosic biomass hydrolysis [49]. The endoglucanase produced by *M. thermophila* I-1D3b showed maximum activity at 60 °C and pH 4.0. Such result may be checked in Figure S1 in Supplementary information section, with mean values, standard deviations of triplicates, and groupings according to Tukey’s test (*p* < 0.05) expressed as U·g.d.s^− 1^ of initial dry solid substrate.

An increase in enzymatic activity was observed with increasing temperature up to 60 °C, indicating improved catalytic performance under this condition. Regarding pH, the maximum activity at pH 4.0 indicates that the enzyme was more efficient under acidic conditions. Preference for elevated temperature is characteristic of enzymes from thermophilic fungi, in which the greater structural rigidity of proteins above 50 °C contributes to maintenance of catalytic activity. The optimum pH of 4.0 falls within the typical range of fungal cellulases, whose catalytic conditions preferentially occur under acidic conditions, consistent with fungal metabolism on decomposing plant substrates [49, 50].

López-López et al. [51] and Xu et al. [52] characterized endoglucanases from *Thielavia terrestris* and reported an optimum temperature of 50 °C, lower than that obtained in the present study, a difference that may reflect the distinct fungal origins and cultivation conditions employed in each study. Qin et al. [44] described maximum activity at 60 °C for an endoglucanase from *Aspergillus oryzae*, which coincides with the value obtained here. Regarding pH, Casciatori et al. [15] reported an optimum pH of 5.5 for *M. thermophila* I-1D3b, which is higher than that observed in the present study, a difference that may be related to extract composition, substrate type, or the production conditions adopted. Andrade et al. [53] observed optimum pH values between 5.5 and 6.0 and temperatures between 40 and 45 °C for an endoglucanase from *Piromyces finnis*, a difference consistent with the anaerobic mesophilic profile of this species. Boonrung et al. [54] observed an optimum temperature of 50 °C for a xylanase from *M. thermophila*, indicating that different enzyme classes from the same fungus may present distinct optimum ranges.

### Thermal stability

Thermal stability determines the ability to maintain catalytic activity during prolonged use at elevated temperatures, and it is a central criterion in the selection of enzymes for biotechnological applications [55]. In this study, the thermal stability of endoglucanase was evaluated at 60 °C for 24 h, and the residual activity profiles shown in Figure S2 of the Supplementary information section were fitted to the model of Sadana et al. [22], allowing estimation of the thermal inactivation constant (*k*_d_), half-life (*t*_1/2_) and parameter α.

The crude aqueous extract (Figure S2a) showed a *k*_d_ of 0.8983 ± 0.3219 h⁻¹, indicating a higher rate of thermal inactivation. The high dispersion observed for this condition suggests greater experimental variability. In contrast, the precipitated sample resuspended 1:1 (v/v) in sodium acetate buffer at pH 4.0 (Figure S2b) showed a *k*_d_ of 0.3818 ± 0.0770 h⁻¹. The half-life of the crude aqueous extract was 0.8832 h; for the buffered condition, *t*_1/2_ could not be calculated because the residual activity remained above 50% throughout the experimental period, preserving approximately 92% of the initial activity after 24 h.

Karnaouri et al. [56], reported a half-life of only 6.02 h at 60 °C for the MtEG5A endoglucanase from the same species, a value considerably lower than that observed in the present study for the buffered sample (although it was not possible to be calculated, the experiment addresses it would be higher than 24 h). This result indicates that, under the buffered conditions adopted here, the endoglucanase showed higher thermal stability than similar preparations previously described. This greater stability is particularly relevant for industrial applications in the second-generation ethanol chain, in which enzymatic saccharification steps frequently operate at elevated temperatures to increase catalytic efficiency and mitigate risks of microbial contamination.

The lower inactivation constant and the high residual activity of the buffered sample indicate that this condition reduced the rate of thermal deactivation and favored maintenance of catalytic activity throughout the assay. In contrast, the crude aqueous extract showed faster activity loss, especially during the first hours of incubation. Since the molecular mechanisms of inactivation were not directly investigated in the present study, interpretations related to aggregation, oxidation, or proteolysis should be regarded only as hypotheses. Xu et al. [52] showed that endoglucanases from thermophilic fungi may exhibit high optimum activity but variable thermal stability depending on the enzyme evaluated. Andrade et al. [53] demonstrated that changes in the structural context or enzyme environment directly affect thermal resistance, reinforcing that extract-preparation conditions, such as buffering, play a decisive role in the observed enzymatic stability.

### Storage stability

Stability during storage directly influences the maintenance of the catalytic activity of enzymatic preparations over time and is therefore a determining factor for their preservation and application [57]. Figure S3 in Supplementary information section shows the residual activity behavior of the endoglucanase precipitated at 70% ammonium sulfate saturation and stored under different temperature conditions over 180 days.

In general, the enzyme showed good initial stability, maintaining residual activity above 70% up to 60 days of storage under the evaluated refrigerated and frozen conditions. After this period, maintenance of enzymatic activity became more dependent on storage temperature, with a more pronounced reduction under room-temperature storage. Low temperatures favor the structural and functional preservation of enzymes during storage, as reported by Souza Lima et al. [58] and Santos Gomes et al. [40].

At the end of 180 days, no significant differences were observed among the refrigerator, freezer, and ultra-freezer conditions according to Tukey’s test at the 5% significance level, indicating that all three conditions were equally effective in preserving residual activity. Although freezing is often associated with greater biomolecule preservation, refrigeration showed performance equivalent to the other low-temperature conditions for the endoglucanase evaluated here, with the additional advantages of lower energy demand, lower operating cost, and simpler infrastructure.

The room-temperature condition showed the greatest loss of activity over time, indicating lower preservation capacity in the absence of thermal control. This behavior is consistent with the literature, which indicates that enzyme stability outside refrigerated conditions depends on specific structural characteristics and tends to be limited over time [59–61]. Therefore, the results indicate that refrigerated storage is the most suitable condition for preserving the enzymatic extract obtained in this study, as it combines satisfactory stability, operational simplicity, and better economic feasibility.

## Conclusions

This study demonstrated that endoglucanase production by *Myceliophthora thermophila* I-1D3b under solid-state fermentation, using sugarcane bagasse and wheat bran as substrates, is technically feasible and reproducible in a multilayer packed-bed bioreactor operated under both batch and continuous regimes. Continuous operation reduced intrabed heterogeneity and promoted greater uniformity of enzymatic activity among the fermentation modules without compromising the average production level relative to batch operation. The thermal profiles remained close to the optimum range for the species, with maximum temperatures of 48.2 °C (batch) and 48.9 °C (continuous), indicating that continuous operation did not impair system stability. Among the concentration strategies evaluated, ammonium sulfate precipitation showed superior performance, with a recovery yield of 78.7% and an activity of 156 U·g.d.s^− 1^, whereas ethanol led to a net loss of activity under the tested conditions. The precipitated extract showed high thermal stability, retaining approximately 92% of its activity after 24 h at 60 °C. Taken together, the results demonstrate that the integration of thermal control, percolation extraction, and downstream processing is critical to the robustness and applicability of the developed platform for the in situ production of cellulases in the second-generation ethanol chain. Future studies should validate these conditions at pilot scale and investigate complementary purification strategies and the economic feasibility of the process.

## Supplementary Information

Below is the link to the electronic supplementary material.

Supplementary Material 1

## Data Availability

The data are available upon request.
