# Supplementary material for: Integrated cellulase continuous production and downstream processing using a packed-bed bioreactor for solid-state fermentation by thermophilic fungus
Source: Bioprocess Biosyst Eng. 2026 Jun 29;49(7):1939–57. doi: 10.1007/s00449-026-03371-1 (PMC13379428; doi:10.1007/s00449-026-03371-1)
Supplement: Supplementary file 1 — Supplementary Material 1 [file 449_2026_3371_MOESM1_ESM.docx]

**Integrated cellulase continuous production and downstream processing using a packed-bed bioreactor for solid-state fermentation by thermophilic fungus**

Nilton S.C. Mafra^a^ and Fernanda P. Casciatori^ab*^

^a^Graduate Program of Chemical Engineering, Federal University of São Carlos, Rod.

Washington Luís km 235 – SP-310, 13565-905 São Carlos, SP, Brazil

^b^Chemical Engineering Department, Federal University of São Carlos, Rod.

Washington Luís km 235 – SP-310, 13565-905 São Carlos, SP, Brazil

***Corresponding author**

Phone: +55 16 3306-6892

E-mail: [fernanda.casciatori@ufscar.br](mailto:fernanda.casciatori@ufscar.br)

https://orcid.org/0000-0001-9274-1241

**Supplementary information section**

**Fig. S1** Effect of pH and temperature on enzymatic activity. * Means that do not share a common letter are significantly different according to Tukey's test (*p* < 0.05)

| (a) |
| --- |
|  |
| (b) |
|  |

**Fig. S2** Thermal stability profiles of endoglucanase at 60 °C for 24 h, fitted by nonlinear regression to a first-order kinetic model: (a) crude aqueous extract; (b) precipitated extract diluted 1:1 (v/v) in sodium acetate buffer (pH 4.0)

**Fig. S3** Residual endoglucanase activity of the concentrated extract (%) under different storage conditions (refrigerator, freezer, room temperature, ultra-freezer, and ultra-freezer with protectant) over 180 days. Residual activity was expressed relative to the control sample, which presented an initial activity of 147.07 ± 2.45 U·g.d.s⁻¹ (considered as 100% activity). Bars represent mean ± standard deviation (n=3). Different letters above the columns indicate significant differences among conditions for the same time interval according to Tukey’s test (p < 0.05)
